# Yerba Mate Extract in Microfibrillated Cellulose and Corn Starch Films as a Potential Wound Healing Bandage

**DOI:** 10.3390/polym12122807

**Published:** 2020-11-27

**Authors:** Meysam Aliabadi, Bor Shin Chee, Mailson Matos, Yvonne J. Cortese, Michael J. D. Nugent, Tielidy A. M. de Lima, Washington L. E. Magalhães, Gabriel Goetten de Lima

**Affiliations:** 1 Department of Paper Sciences and Engineering, Gorgan University of Agricultural Sciences and Natural Resources, Gorgan 00386, Iran; meysam.aliabadi@gmail.com; 2 Materials Research Institute, Athlone Institute of Technology, N37 HD68 Athlone, Ireland; b.schee@research.ait.ie (B.S.C.); y.cortese@research.ait.ie (Y.J.C.); mnugent@ait.ie (M.J.D.N.); tieli.lima@gmail.com (T.A.M.d.L.); 3 Embrapa Florestas, Colombo 00319, Brazil; mailsondematos@gmail.com (M.M.); washington.magalhaes@embrapa.br (W.L.E.M.); 4 Programa de Pós-Graduação em Engenharia e Ciência dos Materiais—PIPE, Universidade Federal do Paraná, Curitiba, Paraná 19011, Brazil

**Keywords:** *Ilex paraguariensis* A. St.-Hil, MFC, wound regeneration, antioxidant, antibacterial

## Abstract

Microfibrillated cellulose films have been gathering considerable attention due to their high mechanical properties and cheap cost. Additionally, it is possible to include compounds within the fibrillated structure in order to confer desirable properties. *Ilex paraguariensis* A. St.-Hil, yerba mate leaf extract has been reported to possess a high quantity of caffeoylquinic acids that may be beneficial for other applications instead of its conventional use as a hot beverage. Therefore, we investigate the effect of blending yerba mate extract during and after defibrillation of *Eucalyptus sp.* bleached kraft paper by ultrafine grinding. Blending the extract during defibrillation increased the mechanical and thermal properties, besides being able to use the whole extract. Afterwards, this material was also investigated with high content loadings of starch and glycerine. The results present that yerba mate extract increases film resistance, and the defibrillated cellulose is able to protect the bioactive compounds from the extract. Additionally, the films present antibacterial activity against two known pathogens *S. aureus* and *E. coli*, with high antioxidant activity and increased cell proliferation. This was attributed to the bioactive compounds that presented faster in vitro wound healing, suggesting that microfibrillated cellulose (MFC) films containing extract of yerba mate can be a potential alternative as wound healing bandages.

## 1. Introduction

The field of wound healing has advanced fast in recent years [1,2], and it is now possible to use films that can protect the humid environment of the wound, be antibacterial, decrease the scar size, and target deliver specific compounds in order to increase the regeneration of the tissue [3]. However, production of these films with incorporation of specific compounds can be time consuming and expensive [4].

Many natural polymers have been reported for wound healing applications such as chitosan/gelatin—anti-inflammatory and wound healing properties [5], alginates—hemostatic properties in exudation/bleeding wounds [6], and dextran—used in chronic wound dressing [7]; this is mainly because these polymers, which are biocompatible, biodegradable and nontoxic, have an organized structure which helps with cell viability and tissue ingrowth and can mimic the extracellular matrix [8]. However, their extraction synthesis in order to obtain a pure raw material involves a lot of steps and can increase their overall costs [9]. Alternatively, microfibrillated cellulose has been reported to be a potential alternative for wound healing applications, since it is very cheap to produce [10,11], very resistant [12] and is able to target deliver drugs for weeks, depending on the affinity of the drug with the cellulose chains [13].

Microfibrillated cellulose, which is commonly obtained by defibrillation of cellulose from kraft paper, produces a homogeneous, transparent, and highly resistant film. In addition to its good adhesion and moisture retention [10], with a similar healing rate compared to bacterial cellulose films [5], but actually presenting a better interaction within the wound [10]. The mechanical defibrillation that occurs from an ultrafine grinding machine enables the incorporation of different compounds during defibrillation [14]. This process heavily affects the interaction between the nanofibrils and the added compounds [12,14]. For instance, previous work has shown that the contact angle, antioxidant, and transparency of the film were severely modified if tannin was incorporated during the defibrillation of cellulose [15]. Therefore, it confers a wide range of opportunities that have not yet been deeply tested within the film synthesis, and the nanosuspension produced during defibrillation of cellulose in any solvent may provide an alternative approach for the incorporation of extract as the solvent. Furthermore, replacing the solvent with the extract may possibly produce a better interaction than if blended after suspension synthesis.

One of the many disadvantages of mixing these extracts within cellulose films is their potential decrease/degradation in bioactive compounds after film formation [16], and blending during defibrillation may protect its compounds, providing a better efficiency in any suitable application.

Yerba mate (*Ilex paraguariensis* A. St.-Hil) is an important native plant, the leaves of which are widely utilized in the southern part of the South American region [17] due to its beneficial compounds and caffeine, and is consumed as a hot beverage, tea [18]. However, recent works have been shown that this plant contains increased contents of caffeoylquinic acids [16,19], to which are attributed the plant’s anticancer [20], anti-inflammatory [21] and antioxidant properties [22]. In addition, yerba mate is within a small number of species in nature that contain these compounds in great abundance [19]. Therefore, the extract from its leaves may have potential applications not yet studied and may also present enormous benefits; hence, wound healing may be a potential application, while cellulose films may be enhanced when containing the aforementioned beneficial properties of this extract.

Films of yerba mate extract are usually synthesized using starch as the matrix for food packaging applications, for which purpose it is a very suitable material [23,24,25]. The incorporation of a high starch content into a defibrillated cellulose containing yerba mate extract may present potential benefits to wound healing, due to its good interaction and it may also decrease its costs [26].

Therefore, the present work investigates the effect of yerba mate extract blended into microfibrillated cellulose films before and after defibrillation and investigates the potential benefits of this material. An increased loading of starch is also incorporated, as well as glycerine, in order to verify the integrity and cellular viability of the film for wound healing applications. 

## 2. Materials and Methods

### 2.1. Materials

Materials used in this work were immortalized human keratinocytes (HaCaT) cell line (Caltag MedSystems Ltd., Buckingham, UK) and NIH 3T3 cell line purchased from ATCC; bleached eucalyptus kraft pulp (Suzano Papel e Celulose, São Paulo, Brazil) for microfibrillated cellulose (MFC) preparation. Corn starch (Rhoster Ltd., Araçoiaba da Serra, Brazil) and Glycerine (Dinâmica química contemporânea Ltd., Indaiatuba, Brazil), *E. coli* from ATCC 25922 and *S. aureus* NCTC 12981.

### 2.2. Film Formation

#### 2.2.1. Yerba Mate Collection and Extract

*Ilex paraguariensis* A. St. Hil. leaves were collected at EMBRAPA Florestas orchard, in which around 10 leaves were plucked from each tree, at random without a specific preference for the age of the leaves. The samples were dried in a microwave oven (Electrolux MEF41, power 1150 W, frequency 2450 MHz) for approximately 3 min, and turned every minute for more efficient drying until the leaves were crisp.

The dried leaves, 20 g, were added to a sealed filter paper cartridge and submitted for extraction using a Soxhlet apparatus with distilled water after extraction. The extract obtained had a solids content of 3.36 g/100 mL.

#### 2.2.2. Starch Solution

Starch (60 vol%) powder was dissolved in either distilled water or yerba mate extract, and was plasticized at 60 °C for 1 h with continuous stirring [27]. For samples containing glycerine, it was also added as 37 wt% of starch.

#### 2.2.3. Microfibrillated Cellulose Gel

The methodology follows a previous work [14]. It consists of adjusting the bleached kraft pulp to 3 wt%/v (3 g/100 mL) using either distilled water, or the previously made starch or yerba mate extract solution (D), and was further fragmented using a 450 W blender for 10 min. Afterwards, it was subjected to further grinding using a Super Masscolloider (Masuko Sangyo Co. Ltd., Kawaguchi, Japan). The function of this mechanical defibrillation is that it promotes the exposure and opening of surfaces that were hidden inside the cellulose material, commonly named as fibrils/microfibrils. This surface modification generates a greater contact area and binding with the microfibrils, leading to an increase in the resistance of the material [28]. These cellulose fibers are of nanometric size (0.1 and 100 nm) on which the stones from the mechanical defibrillation apparatus induce abrasive forces, obtaining a nanosuspension formulation with a gel-like characteristic [29]. Technical parameters are 1500 rpm rotation: 30 steps and 0.1 mm distance between stone disks. Thus, two sets of gels were obtained—a pure MFC using distilled water as solvent, and another set using the yerba mate solution or starch. In order to plasticize the films, glycerine was added as 37 wt%/wt of cellulose (1.11 g glycerol/100 mL of gel solution).

#### 2.2.4. Solvent Evaporation Casting Film Formation

In order to form the films, 40 mL of each gel (diluted in a 1/4 ratio of distilled water) was poured on petri dishes, which were immediately dried at 60 °C using an Q319V Vacuum Drying Oven (Quimis, Diadema, Brazil). The samples used in this work follows the denomination of the material used; therefore, MFC for microfibrillated cellulose, STC for starch, YM for yerba mate extract (20% as the pure extract, and 10% as the extract diluted by half) and GLY for glycerine.

For films containing the yerba mate after defibrillation of cellulose (A), the pure (6%) MFC suspension was diluted into the yerba mate extract in order to obtain the sample A-MFC-YM10%.

### 2.3. Fourier Transform Infrared Spectroscopy (FTIR)

The microstructure was investigated using the FT-IR technique on a Perkin Elmer spectrometer (FT-IR/NIR Frontier, PerkinElmer, Waltham, UK) using an attenuated total reflectance (ATR) accessory with a zinc selenide (ZnSe) crystal surface. A resolution of 4 cm^−1^ and arithmetic average of 64 scans was used in the wavenumber range of 4000–550 cm^−1^.

### 2.4. Thermo and Mechanical Analysis

For Differential scanning calorimetry (DSC), film weights around 9–12 mg were encapsulated in alumina sample pans. A temperature ramp from 0 °C to 400 °C at a rate of 10 °C/min was used with an empty closed alumina pan as a reference. TGA curves were obtained with a heating rate of 10 °C min^−1^ until 600 °C using platina pans (TA Instruments, New Castle, PA, USA) with samples weighing around 3.0 mg. The experiments were carried out under nitrogen flow of 50 mL min^−1^, in a Q600 SDT—TA Instruments (TA Instruments, New Castle, PA, USA) for both DSC and TGA analysis.

Dynamic Mechanical Analysis (DMA) analyses were performed on DMA Q800 TA Instruments (TA Instruments, New Castle, PA, USA) equipment using the film tension clamp. Stress−strain tests with ramp force of 1 N/min up to 18 N. All tests from DMA were performed using three scans per sample. The mean stress−strain for each film condition for the plot for each film.

### 2.5. Yerba Mate Extract Release

Release performed at 25 °C, 0.100 g of film in 50 mL of DI water. The released concentration was determined by UV (250–200 nm), at time points 0, 1, 3, 5, 7, 24 and 48 h, against a Partial least squares (PLS) model in the same region (R^2^ prediction: 0.992, with a prediction error of 2.9 × 10^−4^). The PLS was constructed using yerba mate extract solutions (0 to 0.015% (wt./vol.).

### 2.6. Antioxidant Activities (DPPH and ABTS)

Antioxidant activity assays were performed in a spectrophotometer UV/VIS (Shimadzu Corp., model 1800, Kioto, Japan). The antioxidant capacity of extracts by free radical DPPH (2,2-diphenyl-1-picrylhydrazyl) was obtained by a procedure from a previous work [30] with minor modifications. First, 25 mg of each film sample was extracted in 3 mL of distilled water, for use as a film extract solution. Then, 0.1 mL of the extract solution was added to 3.9 mL of DPPH methanolic solution (60 μmol L^−1^) and kept in the dark for 30 min (reaction) and the absorbance was measured at 515 nm. The results were expressed as a percentage of radical scavenging.

The free radical scavenging by ABTS radical was determined using the procedure of a previous work [31]. A volume of 88 µL of potassium persulfate (140 mmol/L) was added to 5 mL of ABTS (7 mmol/L). The mixture was stored in an amber bottle in the dark at room temperature for 16 h. The ABTS radical solution absorbance was adjusted at 0.70 ± 0.05 at the 734 nm in spectrophotometer. Then, 0.3 mL of film extract solution was added to 3.7 mL of de ABTS radical for 10 min in the dark. After this, the absorbance was measured at 734 nm. The results were expressed as a percentage of radical scavenging.

### 2.7. Antibacterial Evaluation

The antibacterial activity of the samples was investigated with the shake flask method and disk diffusion method. For the disk diffusion, samples were cut into 6 mm discs which were then sterilized by UV light. Mueller−Hinton Agar plates were inoculated with 100 µL of either *E. coli* ATCC 25922 or *S. aureus* NCTC 12981 at a density of 1 × 10^8^ CFU/mL which was dispersed over the entire agar surface with a sterile glass L-shaped spreader. Triplicate discs were aseptically placed on each plate before incubation at 37 °C overnight. After incubation, the diameter of each zone of inhibition observed was measured and recorded. 

For the shake flask method, the bacteria were first incubated in Luria–Bertani medium (LB medium, 1% peptone, 0.5% meat extract, and 1% NaCl, pH 7). The inoculation was then conducted at 37 °C for 24 h under shaking and the obtained bacterial suspension was diluted with the previous peptone medium solution. Afterwards, 0.1 mL of diluted bacteria suspension was cultured in 10 mL liquid peptone medium and 50 mg of sample was added to the bacterial suspension. The inoculated medium was incubated at 37 °C for 24 h under shaking. After incubation, the antibacterial activity was monitored by measuring the optical density *O.D.* of the culture medium at 620 nm and calculating the inhibition percentage using the following Equation (1):

(1) $$\frac{O.D.\;bacteria\;–\;O.D.\;sample}{O.D.\;\;bacteria}\;\times 100$$

### 2.8. Cytotoxic Evaluation

All samples were sterilized using ethanol 70% for 30 s, phosphate buffered saline (PBS) for 30 s and followed by DMEM media for 30 s prior to cytotoxic evaluation following the methodology of a previous report [32]. 

#### 2.8.1. Elution Assay (Cytotoxicity Testing)

A sample of 100 µL of HaCaT cells (1.5 × 10^5^ cells/mL) was seeded in a 96-well plate and incubated overnight at 37 °C. Two different concentrations of extracts, 5 mg/mL and 25 mg/mL were prepared for each sample using DMEM media. They were incubated for 24 h at 37 °C prior to testing. The following day, the media was removed from the plate and 100 µL of the extract was added into each well and incubated overnight at 37 °C. Following incubation, MTT assay was carried out by treating the cells with 100 µL 0.5 mg/mL MTT solution and incubated for 3.5 h in the 37 °C incubator. The MTT solution was then removed and 100 µL DMSO solution was added into each well. The plate was read at 540 nm using a Synergy HT BioTek Plate Reader. The cell viability was calculated using the following Equation (2):

(2) $$\frac{Abs\;@\;540\;nm\;treated\;cell}{Abs\;@\;540\;nm\;untreated\;cell}\;\times 100$$

#### 2.8.2. Determination of p65-NF-κB Protein

The 5 × 10^5^ cells/well of HaCaT cell line was seeded on the 96-well plate. After overnight incubation at 37 °C, the media was removed and 100 µL of each sample extract at a concentration of 25 mg/mL was added into the well separately and incubated for another 24 h. The amount of total NF-kB p65 and of phosporylated NF-kB p65 proteins presented in the cell lysate was then quantified using an Invitrogen NFkB p65 (Total/Phospho) Human InstantOne ELISA kit, following the manufacturer’s instructions.

#### 2.8.3. Wound Scratch Assay

Some 3T3 cell lines were plated in 24-well plates. The cells were seeded and incubated overnight until it reached a confluence of 70–80%. The next day, a mechanical scratch wound was created by gently scraping with the sterile 10 µL micropipette tip. The cells were rinsed with PBS and treated with test formulation at the concentration of 25 mg/mL. It should be noted that the test formulation was prepared using serum free-DMEM to avoid cell proliferation. The photomicrographs were taken using a digital camera, which was connected to the inverted microscope after 24 h of incubation with test formulation. The scratched area was then measured using ImageJ software. The % wound closure was calculated using the following Equation (3):

(3) $$\%\;wound\;closure=\frac{A_{0}-A_{t}}{A_{0}}\;\times \;100\%$$

where *A*_0_ is the area of the wound measured immediately after scratching and *A_t_* is the area of the wound measured 24 h after the scratch was performed [33].

#### 2.8.4. Statistical Analyses

The data, where applicable, were submitted to descriptive statistical analysis. Statistical analyses were performed using the analysis of variance (ANOVA). For comparison between groups, the Tukey HSD (“Honestly Significant Difference”) post hoc test was used.

## 3. Results and Discussion

### 3.1. Characteristics of Blending the Extract with MFC at Different Stages

#### 3.1.1. Microstructure

**Figure 1:**
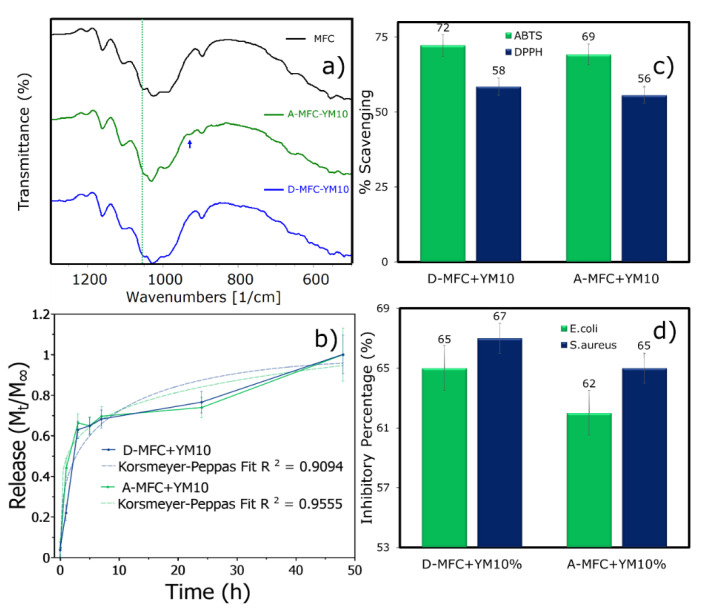
Studied samples for microfibrillated cellulose (MFC) films containing yerba mate extract blended during (D) the defibrillation process and after (A), (**a**) FTIR spectra, where dashed lines indicate the most important region and the arrow is an increased peak found after the extract is blended. (**b**) YM extract release and best-fit using Korsmeyer−Peppas, (**c**) ABTS and DPPH antioxidant scavenging activity and (**d**) bacteria growth inhibition of 25 mg samples containing yerba mate extract.

In order to obtain the best possible interaction between the yerba mate extract with the MFC, blending was performed in two steps: during defibrillation of the cellulose (D), and after defibrillation (A). The first indication that variation indeed occurred in the structure, depending as to when blending was performed on the samples, was exhibited by the FTIR. Only the most important region is shown, in order to perceive the differences of blending steps (Figure 1a). The region for MFC within 1200–1105 cm^−1^ was characteristic of C–O–C symmetric and asymmetric groups [34]; 1026 cm^−1^ and 896 cm^−1^ corresponding to C–O cellulose backbone and C–H in cellulose, respectively [35]. 

The band 1051 cm^−1^ was assigned to cellulose primary alcohol C–OH stretch [36], a region also assigned to polyphenols from the pure yerba mate extract (1040–1050 cm^−1^ ) [37]. The cellulose band in this region (dashed lines from Figure 1), was the most prominent one in the cellulose spectra that was not shown when the extract was blended after defibrillation of MFC. A heavy decrease in this band indicated an oxidation from the bioactive compounds found within the yerba mate extract. Flowing oxygen is known to oxidize extracts of leaves, such as green tea, and if the surface area of this solution was readily exposed, it may have contributed to a higher oxidation effect [38]. It is possible that blending the extract during defibrillation, by ultrafriction grinder, could have a better interaction within the nanofibrils, as previously reported for tannins and MFC [39], so these nanofibrils could potentially protect the bioactive compounds. Nonetheless, when the blending was performed after cellulose defibrillation, a slight increase could be seen at 926 cm^−1^ attributed to yerba mate extract [40].

#### 3.1.2. Yerba Mate Extract Release, Antioxidant, and Antibacterial Analysis

The release of films containing yerba mate extract (YME) blended during and after defibrillation exhibited few differences (Figure 1b), though there was a better fit for Korsmeyer−Peppas release after defibrillation, indicating that the interaction of the extract with the cellulose microfibrils had altered, in agreement with the microstructure investigation results. 

The antioxidant nature of yerba mate makes it able to scavenge free radicals from biological systems that may be associated with numerous degenerative conditions [20,22]. In this specific case (Figure 1c), this test evaluated its power by measuring the decrease in the absorbance of DPPH at 515 nm—polyphenols in the film were measured with a solution of 0.1 mL that contained 25 mg of film which was extracted in 3 mL of solvent and was able to reduce up to 65% from a 2.9 mL solution that contained 24 mg/L of DPPH. This concentration of extract (10%) has also been reported by other authors to be effective in its scavenging ability [41]. The antioxidant power after the reaction of ABTS with yerba mate extract was also measured at 734 nm, presenting scavenging values of ~70%. Therefore, yerba mate in the films can still remain active as an antioxidant. 

When yerba mate is incorporated into films, such as starch, it has been reported to decrease DPPH activities when dried for film formation [23], and has also been reported to exhibit low activity values [25]. However, the values presented herein were somewhat high and there was a slight increase, not significant, in antioxidant activity when blending was performed during defibrillation of cellulose. These antioxidant activities from yerba mate extract have been reported to be due to chlorogenic acid’s caffeoylquinic acids (3-CQA, 4-CQA, 5-CQA) and dicafeoylquinic acid (3,5-DQA) [42]. The microwave protected the degradation of these compounds when drying the leaf [43] by inactivating the enzyme to a potential oxidation. These compounds found in the extract have been reported to also present hepatoprotective [44] and anticancer [45] properties and attenuate symptoms of inflammatory diseases [46] which act through a mechanism known as oxidative stress [47].

Finally, films containing yerba mate were able to inhibit common pathogens, *E. coli* and *S. aureus*, in the shake flask method (Figure 1d); however, no inhibition occurred for disk diffusion (Figure S1 in the Supplementary Material). We attribute this effect to the mechanism of yerba mate polyphenol release, in which the agitation system from within a solution containing bacteria will induce an improved release over a diffusion methodology. Inhibition of both bacteria has been reported for yerba mate extracts in both methodologies for antibacterial testing [48]. For the shake flask method, films blended with the extract during MFC production had slightly increased values, but not significant.

#### 3.1.3. Mechanical and Thermal Properties

**Figure 2:**
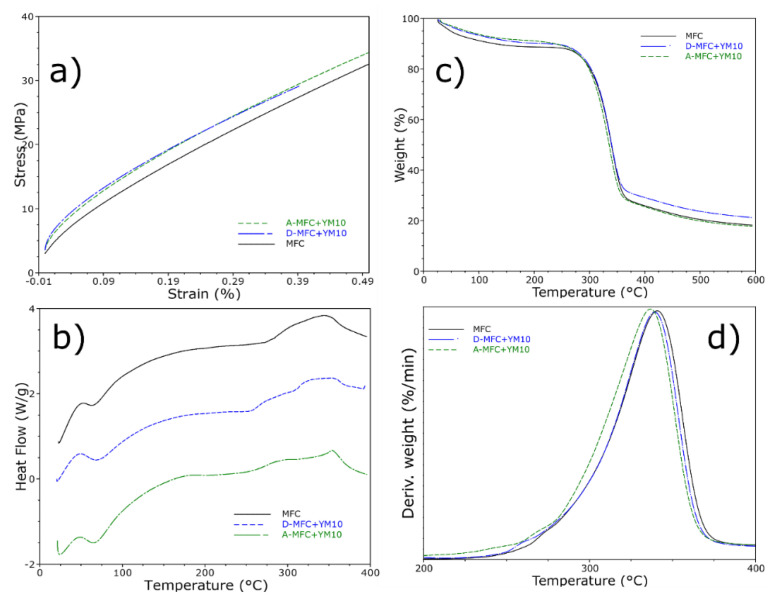
(**a**) Stress−strain curves for tensile tests in DMA, (**b**) differential scanning calorimetry and (**c**) thermogravimetric analysis with its (**d**) first derivative for the MFC films.

Films with defibrillated cellulose from kraft pulp are known to be very resistant [10,29], and this can be improved if crosslinked with other materials—such as chitosan [49]—at the expense of becoming less flexible and more brittle. The mechanical defibrillation used in this work, also reported by other works to obtain nanofibrils [29,39], strongly affected the results of the tensile tests performed by DMA (Figure 2a). In the case of yerba mate, the film slightly increased its resistance, and blending during defibrillation resulted in it becoming more resistant compared with blending afterwards. It is possible that some polyphenols from the yerba mate may have increased the affinity from the cellulose groups, leading to an increased resistance, a behavior already shown before when incorporated into starch films [41]. In addition, these films present high tensile resistance and are reported to obtain an elastic tensile modulus in the order of ~ 5 GPa [12,14], much higher than those obtained by acid hydrolysis or chemically processed nanocellulose from agri industry residues such as sunflower hulls [50].

Films containing yerba mate extract were subjected to thermal evaluation, in order to investigate if any changes may have occurred with the incorporation of the extract (Figure 2b). The differential scanning calorimetry indicated that the addition of yerba mate presented, in the first event, a broad endothermic peak compared to MFC. This first event was attributed to the loss of stored water for pure MFC, and if blending was performed after defibrillation, this event initiated at lower temperatures—indicating a somewhat weak thermal stability. Nonetheless, the addition of yerba mate extract also presented a broader endothermic peak at ~250 °C, which was characteristic of the yerba mate extract (Figure S2 in the Supplementary Material) possibly due to deterioration of polyphenol compounds. Finally, the last peak initiating around 350–380 °C indicated cellulose degradation.

This degradation stage was approximately at 352 °C for pure MFC (Figure 2c,d), presenting only one step, related to cellulose degradation of glycosyl units and oxidation breakdown, in order to form products with a low molecular weight [14]. Furthermore, when the extract was incorporated it seemed to exhibit a better thermal stability at initial temperatures ranging from 0–250 °C, but with a decrease in the maximum temperature of decomposition from cellulose (Figure 2c), possibly due to lower decomposition from the extract compounds (~310 °C, Figure S2 in the Supplementary Material). Nonetheless, blending of the extract during defibrillation had a lower impact compared to blending after defibrillation. 

Microfibrillated cellulose from kraft paper produced by ultrafine grinding has already been reported by our group to present a protective effect [14], where the cellulose crystals delayed thermal degradation and protected compounds that were blended within—presenting a “shielding effect” [12]—attributed to the hemicellulose. The increased residual material, for temperatures higher than 400 °C, from blending the extract during defibrillation of cellulose was another indication of this effect.

Due to the described potential benefits of blending the extract during defibrillation, and because it was also possible to use the whole extract, samples were formed with this methodology while also blending in different components to confer different abilities. 

### 3.2. Characteristics of MFC with Extract Blended with Starch and Glycerine

#### 3.2.1. Microstructure of Blending Various Materials within MFC

**Figure 3:**
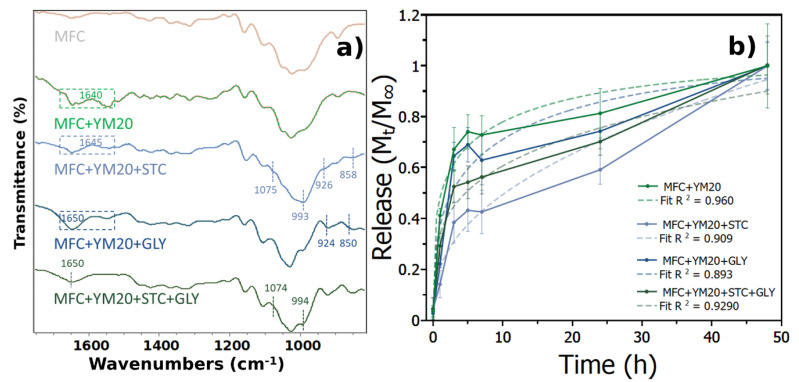
(**a**) Casting films FTIR spectra, assigned numbers signify the formation of new bands by the addition of the specific material. (**b**) Yerba mate extract release with best-fit using Korsmeyer−Peppas correlation for each curve.

Aside from the previously mentioned bands from microfibrillated cellulose, main bands are also shown at 3300–3500 cm^−1^ assigned to O–H stretching and 2900–3000 cm^−1^ which is assigned to C–H stretching (Figure S3 in the Supplementary Material). However, because the structure of MFC and starch is similar, many of its characteristic peaks overlap and it is difficult to perceive any differences if starch is incorporated (Figure 3a). However, and in agreement with previous works, a distinct peak could be seen at 1074 cm^−1^ attributed to starch C–O–H bending and bands at ∼925 cm^−1^, 858 cm^−1^ and 760 cm^−1^ assigned to vibrations of glycosidic ring [41]. 

The addition of glycerine, besides increasing –OH bands due to the increased ability of glycerine to absorb water, also exhibited new bands. Such as 2934 cm^−1^, assigned to aliphatic group, 925 cm^−1^ bound –OH in glycerine, and 851 cm^−1^ stretching vibration of C–O–C groups [51].

The incorporation of the whole extract within the defibrillation of cellulose led to the appearance of new bands in the region around 1640 cm^−1^, that are related to the stretching and bending vibration of hydrogen bonding –OH water groups [52] (squared dashed line on Figure 3), this was to be expected because of the casting synthesis to produce the films involved a large amount of water compared to extrusion [41]. This meant that a strong interaction between MFC and YM occurred with no variations within the region of 800–1200 cm^−1^, similar to what was found for a slower concentration of YM extract if blended with MFC during defibrillation, indicating that no oxidation occurred due to synthesis. 

When starch was added into the films, the region around 1640 cm^−1^ was still present; however, the cellulose region of 800–1200 cm^−1^ shifted to the C–O–H bending of starch due to the high content loading of this material into the film, besides also presenting its characteristic glycosidic starch ring bands. In addition, the C–OH primary alcohol from cellulose was also shown, albeit it was also discussed that it could be related to the polyphenol band, previous works suggested that a shifting also occurred from the polyphenol band (1050 cm^−1^ to the 1020 cm^−1^) in starch with yerba mate extract films [41]. A new band was also perceived at 1074 cm^−1^, assigned to a stretching vibration of the C–O bond in C–O–C groups [52] of starch. Therefore, the addition of starch strongly affected the structure of this film, in which cellulose and yerba mate extract might be grouped within the high content starch chains.

The addition of glycerine exhibited its characteristic bands that were perceived without YM extract, 924 cm^−1^, 850 cm^−1^, an increase in –OH bands and the region around 1640 cm^−1^ was also present. However, when starch was added within the film containing glycerine and extract with MFC, the region from 1640 cm^−1^ only presented one band, with the overall profile similar to glycerine, containing a small shift to starch at 994 cm^−1^ and a new small band around 1074 cm^−1^. Therefore, the overall spectra from FTIR confirmed the incorporation of all products to the films by identifying the characteristic bands of each compound.

#### 3.2.2. Extract Release, Antibacterial and Antioxidant Activities

The yerba mate extract release rate exhibited a fast release in the first seven hours and a continuous release afterwards, which continued even after 48 h, the last time point not shown in the curve, (Figure 3b). The initial release presented the same increase profile, though for samples containing glycerine there was a slight increase in drug release compared to the other samples which may be related to the plasticizer effect that disrupted the hydrogen bonds within the material [53]. The release profile was similar to a previous work that blended the extract with nanocellulose [54]. 

Polymeric films are very useful for wound healing, and the most important aspect may be their ability to maintain a humid environment, while also loaded with compounds containing antibacterial properties that can prevent infection [3]. Yerba mate is known to be antibacterial [55], but it is important that this extract is delivered in a controlled manner otherwise it will not be effective [56]. Ideally, the drug should also be delivered over long periods of time so as not to need frequent changes of dressing. In this work, yerba mate had a very fast release in the first three hours (Figure S4 in the Supplementary Material)—achieving 65% of drug release in samples containing only MFC, while with glycerine and starch it had a release of ~60 and 30%, respectively. Nonetheless, all samples had fully released the extract after 48 h. Even though this is considered a rather quick release compared to hydrogels produced with micelles [57], which can release drugs for weeks, it is important to remember that these samples were produced by solvent-evaporation casting and present standard release values [58]. 

However, no significant differences were perceived when glycerine was added, contrarily to when starch was in the structure. These differences may be attributed to the variation in the structure found within the FTIR. Nonetheless, these films were able to release yerba mate at specific dosages, and the variation on the release profile after seven hours may be attributed to the relaxation of macromolecular chains which presented a low fit for Korsmeyer−Peppas release.

**Figure 4:**
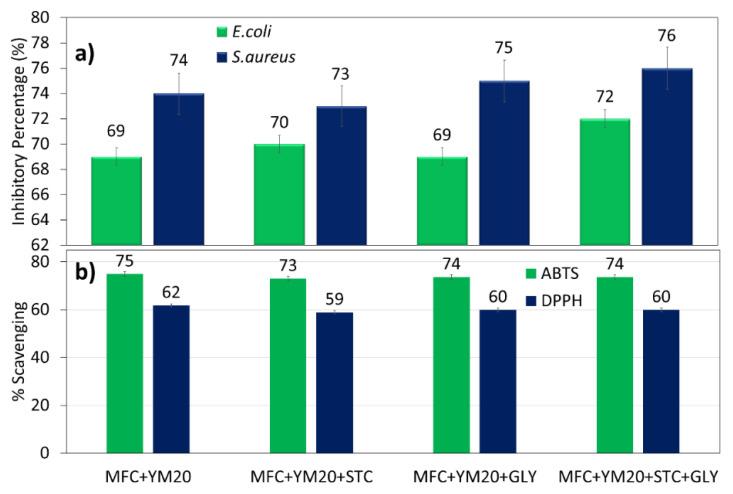
(**a**) Antibacterial activities of the studied MFC films containing yerba mate extract and (**b**) antioxidant activities for ABTS and DPPH.

Yerba mate extract incorporated into the films presented, for all samples, antibacterial activity (Figure 4a) using the shake flask method, against *E. coli* and *S. aureus*, with activity over 70%, values slightly higher than samples containing diluted extract (Section 3.1.2), albeit not statistically significant. However, they did not present antibacterial activity for the disk diffusion methodology (Figure S5 in the Supplementary Material). It is possible that the polyphenols did not spread within the medium from disk diffusion, whereas with agitation they were properly mixed by an external force, and this may be the reason for the result on antibacterial tests. However, it may also be due to the UV performed before test, that may have crosslinked the material, or oxidated the polyphenols, and decreased their effectiveness by inhibiting their diffusion out of the films which resulted in the absence of halo (Figure S5 in the Supplementary Material).

As already described, yerba mate is known to be effective against these two pathogens and works have suggested its usage in foods as a preservative [59]. A recent study tested a wide range of Gram-positive and Gram-negative bacteria against yerba mate, and it suggested that its mode of action was independent from classical bacterial resistance mechanisms [55], attributed to its polyphenol compounds. In addition, there was greater inhibition of this extract against Gram-positive compared to Gram-negative, in agreement with this work [48]. 

**Figure 5:**
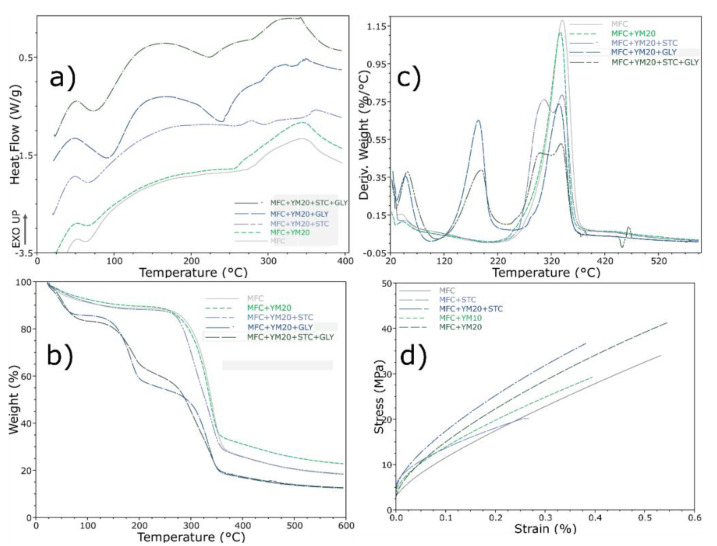
(**a**) Differential scanning calorimetry and (**b**) thermogravimetric analysis with its (**c**) first derivative for the films containing yerba mate extract and (**d**) stress−strain curves for MFC films performed by DMA.

Antioxidant activities of the films containing the extract had no significant differences and presented values higher than 70% for ABTS and 60% for DPPH (Figure 5b). Therefore, the incorporation with MFC, starch and glycerine did not reduce its potential to scavenge free radicals. Such results are in agreement with other works that used starch to incorporate the YM extract [60], also with a synthesized polysaccharide obtained from the YM leaves [60]. The polyphenols in the films could still remain active as antioxidants in order to scavenge free radicals, and might also contribute to wound healing processes by trapping free radicals and inhibiting the oxidative stress that results in tissue damage [61].

#### 3.2.3. Thermal and Mechanical Properties

Differential scanning calorimetry of the studied films indicated that the addition of YM did not vary much from the pure MFC film (Figure 5a). However, the addition of starch to the system slightly increased the first endothermic event, attributed to the starch gelatinization transition peak temperature [62], and shifted to a higher temperature (68 °C). The same effect occurred in the first endothermic event with the addition of glycerine, in which it reduced the intermolecular forces of the film, while also increasing the mobility of the polymer chains [63]. The glycerol promoted the hygroscopicity of the cellulose films, due to its hydrophilic character [53], and resulted in a strong impact on the thermal stability of these films. 

Thermogravimetric analysis exhibited that yerba mate extract slightly decreased the maximum temperature of decomposition, with a slightly improved thermal resistance around 20–250 °C. The addition of starch presented two main degradation steps that almost overlap; the first one was attributed to starch decomposition (around 305 °C), and the second from the cellulose (340 °C). The yerba mate extract decomposition could be seen at the beginning of this degradation curve. 

The addition of glycerine further decreased the stability, as observed with the DSC results. Three main degradation stages occurred (Figure 5b,c), and the first—from 25–100 °C—was related to the water absorbed; also, a weight loss at 125–240 °C attributed to glycerine, and the biggest, within 250–375 °C, was attributed to cellulose and other compound degradation. Besides these, small events could be seen at 220–270 °C for samples containing yerba mate extract, which further proved the melting features due to bonding.

Dynamic mechanical analysis of the films (Figure 5d), performed in tensile mode as a stress−strain procedure, presented a profile with an elastic linear zone followed by a nonlinear behavior. In addition, it also revealed that the addition of yerba mate slightly increased the resistance of the films for MFC and starch. Previous reports with films containing starch and yerba mate showed a decrease in mechanical resistance with incorporation of the extract [23]; however, no significant variation was reported when it was blended with nanocellulose [64]. Nevertheless, we attributed the positive effects to the MFC that, as described by the FTIR, may protect its compounds, and have intermolecular bonds between nanofibrils and polyphenols. However, addition of the extract to the MFC with glycerine decreased the film resistance (Figure S6 in the Supplementary Material), even if starch was also added to the structure. 

#### 3.2.4. Cell Activity Values

**Figure 6:**
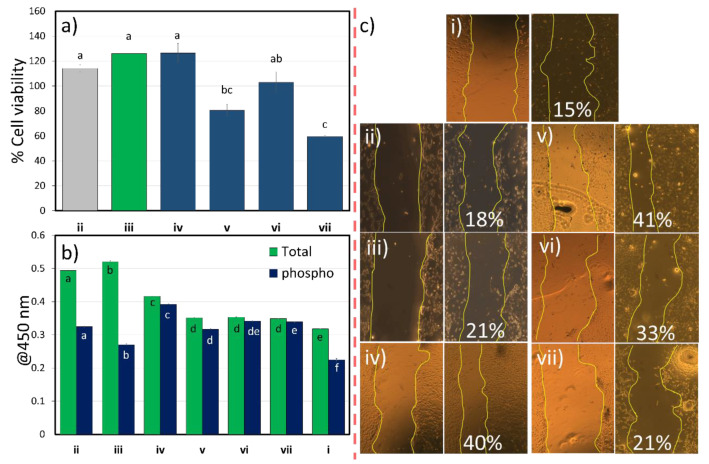
(**a**) Cell viability elution assay gray colour assigned to pure element (MFC), green is addition of YM10 and blue for the various samples containing YM20, (**b**) phosphorylated NF-κB p65 activity and (**c**) wound healing in vitro scratch assay at 25 mg/mL, (i) control, (ii) MFC, (iii) MFC+YM10, (iv) MFC+YM20, (v) MFC+YM20+STC, (vi) MFC+YM20+GLY, (vii) MFC+YM20+STC+GLY; same letters in each composition do not differ by Tukey’s test (*p* < 0.01).

Cell viability of the studied films, for the majority, presented as higher than 70% (Figure 6a), meaning that the films were not cytotoxic. In the case of MFC, it has been shown by our group that it already presents good cell viability results, while also being a cheap method for wound healing applications [10]. When yerba mate was incorporated, although it presented higher values and increased with the extract content, it was not significant. However, the addition of starch significantly decreased the cell viability, meaning that starch did not induce any improvement on its own, similar results have been reported before [65]; nonetheless, still presenting as nontoxic when incorporating the extract. In addition, glycerine did not have a significant effect on cell proliferation, and might cause a reduction/inhibition on cell proliferation [66]. However, only the mixture of the MFC and YM with starch and glycerine caused a significant reduction and low values of cell viability (<60%).

The healing effect could also be perceived by the test with Nuclear factor kappa B (NF-κB) p65 signalling (Figure 6b). This transcription factor is related to the inflammatory process also associated with initiating proinflammatory target gene expression [67]. Nonetheless, NF-κB plays a crucial role in fibroblast healing since it can supervise the expression of genes involved in wound healing processes [68]. Furthermore, it also plays a key role in regeneration of various biomaterials [69,70]. In this case, the 3T3 cells were incubated in contact with the films to stimulate the signalling pathway, and the total protein was determined from the cell lysate using the NF-κB antibody cocktail. The values presented significant increased activity for MFC and MFC-YM10 films, and a significant decrease with an increased content of the YM extract, which maintained the same levels for the other films. 

Compared to control, samples of MFC and low YM extract content may suggest a positive result, but indices of this activity should be kept within normal limits. This is because an overexpression could lead to impaired wound healing [71], which may explain the results found for the scratch assay (Figure 6c), whereas these samples presented lower values from all films for the wound cell wall contraction (18 and 21% compared to control 15%). 

Yerba mate extract has been shown to be good as an anti-inflammatory and to reduce the NF-κb phospolyrated levels [21]. However, in the present work, it did not self-regulate 3T3 cells and this increase has been previously shown to promote faster healing in vitro, related to promoting cell migration and proliferation via the NF-κb signalling pathway [69,70,72]. 

## 4. Conclusions

Microfibrillated cellulose films blended with extract of yerba mate during defibrillation presented increased resistance, high mechanical strength and protected its bioactive compounds during thermal degradation. In addition to that, there was a fast release in the first seven hours of the extract, followed by a gradual release until full release at 48 h. These films were able to present antioxidant activities over 60% for all conditions and antibacterial activity only when compounds were released into the medium by shake flask method against two known pathogens *S. aureus* and *E. coli*. When starch was incorporated, there was an increase in tensile properties and a decrease in thermal stability; while incorporation of glycerine presented a heavy decrease in tensile and thermal behavior due to its plasticizing effect. Furthermore, the addition of yerba mate slightly increased the resistance of the films for MFC and starch, attributed to the interaction from polyphenol extracts with the hydrogen bonding –OH water groups. Nonetheless, they were still able to present antioxidant activities over 60% and antibacterial activity only in the shake flask method when the extract was incorporated within the structure. Lastly, cell viability presented that the majority of the films were nontoxic, and that yerba mate increased the regeneration by the scratch assay in vitro. However, starch presented significantly lower cytotoxic values, though only considered toxic when incorporated with glycerine, but samples containing increased concentrations of yerba mate extract were able to increase the wound healing in vitro from up to 40% for samples containing MFC and starch with the whole extract in 24 h and increased phosphorylation. Therefore, alternative approaches for using the extract, such as for wound healing, may be feasible when producing this film. 

## Supplementary Materials

The following are available online at https://www.mdpi.com/2073-4360/12/12/2807/s1, Figure S1. Antimicrobial disk diffusion test macroscopic images for bacteria inhibition after 24 h for *S. aureus* (a) and *E. coli* (b) where (i,iii) A-MFC+YM10, (ii,iv) D-MFC+YM10, Figure S2. Thermogravimetric analysis with its first derivative for the yerba mate extract, Figure S3. FTIR of the raw materials, pure MFC, MFC blended with starch (MFC+STC) and MFC blended with glycerine (MFC+GLY), Figure S4. Yerba mate extract release in the initial release of three hours with best-fit using Korsmeyer−Peppas correlation for each curve, Figure S5. Antimicrobial disk diffusion test macroscopic images for bacteria inhibition after 24 h for *S. aureus* (a) and *E. coli* (b) where (i,ii) MFC+YM20, (iii,iv) MFC+YM20+STC, (v,vi) MFC+YM20+GLY, (vii,viii) MFC+YM20+STC+GLY, Figure S6. Stress−strain for the MFC films containing glycerine by DMA.
