# Supplementary material for: Yerba Mate Extract in Microfibrillated Cellulose and Corn Starch Films as a Potential Wound Healing Bandage
Source: Polymers (Basel). 2020 Nov 27;12(12):2807. doi: 10.3390/polym12122807 (PMC7761128; doi:10.3390/polym12122807)
Supplement: Supplementary file 1 [file polymers-12-02807-s001.pdf]

Supplemental Information

Yerba mate extract in Microfibrillated cellulose and Corn Starch films as a potential wound healing bandage

Meysam Aliabadi [1], Bor Shin Chee [2], Mailson Matos [3], Yvonne J. Cortese [2], Michael J. D. Nugent [2], Tielidy A. M. de Lima [2], Washington L. E. Magalhães [4], Gabriel Goetten de Lima [3] *

[1] Department of Paper sciences and engineering, Gorgan University of Agricultural Sciences and Natural Resources, Gorgan, Iran

[2] Materials Research Institute, Athlone Institute of Technology, Athlone, Ireland

[3] Programa de Pós-Graduação em Engenharia e Ciência dos Materiais - PIPE, Universidade Federal do Paraná, Curitiba, Paraná, Brazil

[4] Embrapa Florestas, Colombo, Brazil

* Corresponding authors: ggoetten@research.ait.ie

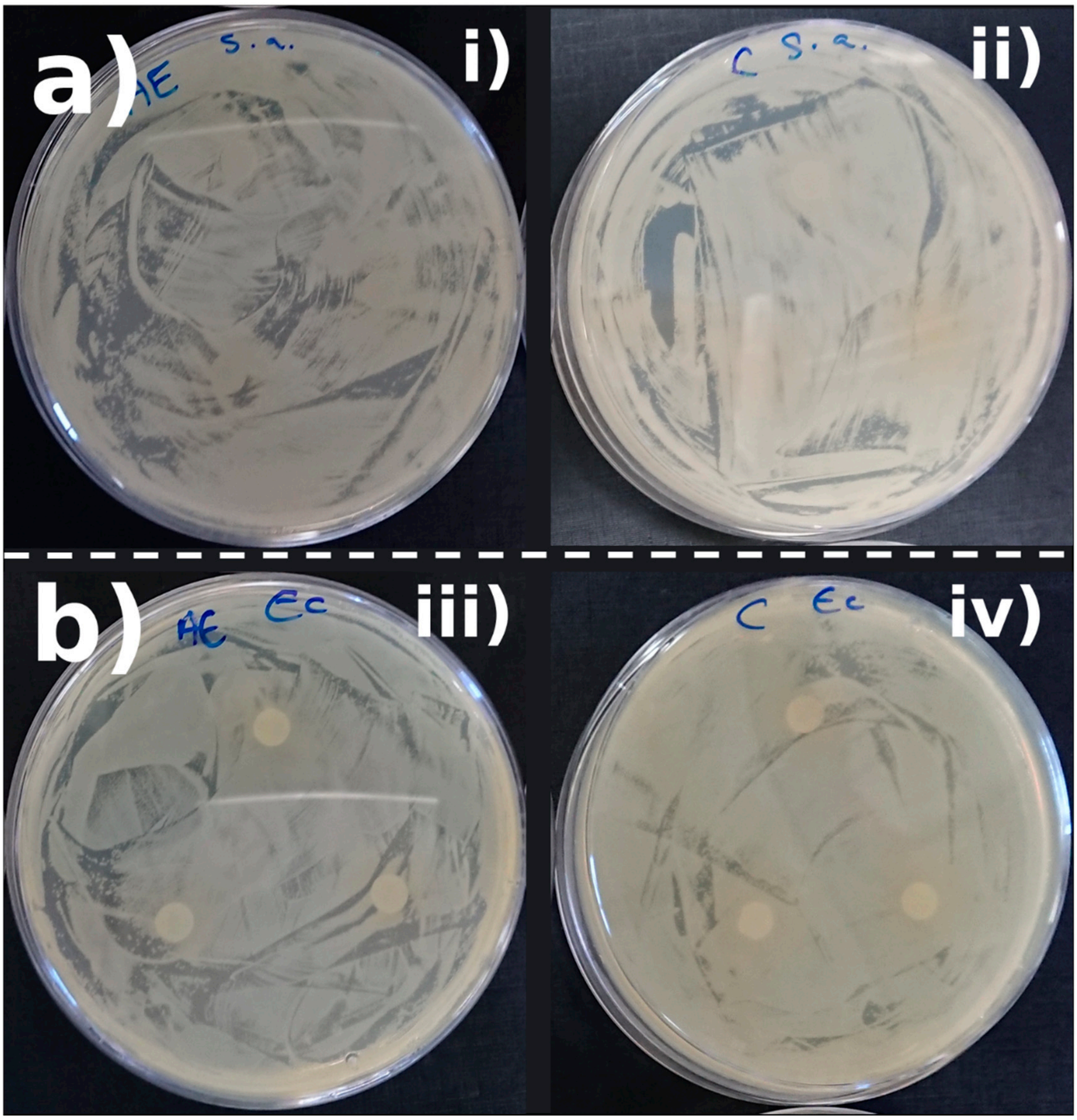

Figure S.1. Antimicrobial disk diffusion test macroscopic images for bacteria inhibition after 24 h for *S. aureus* (a) and *E. coli* (b) where (i,iii) A-MFC+YM10, (ii,iv) D-MFC+YM10

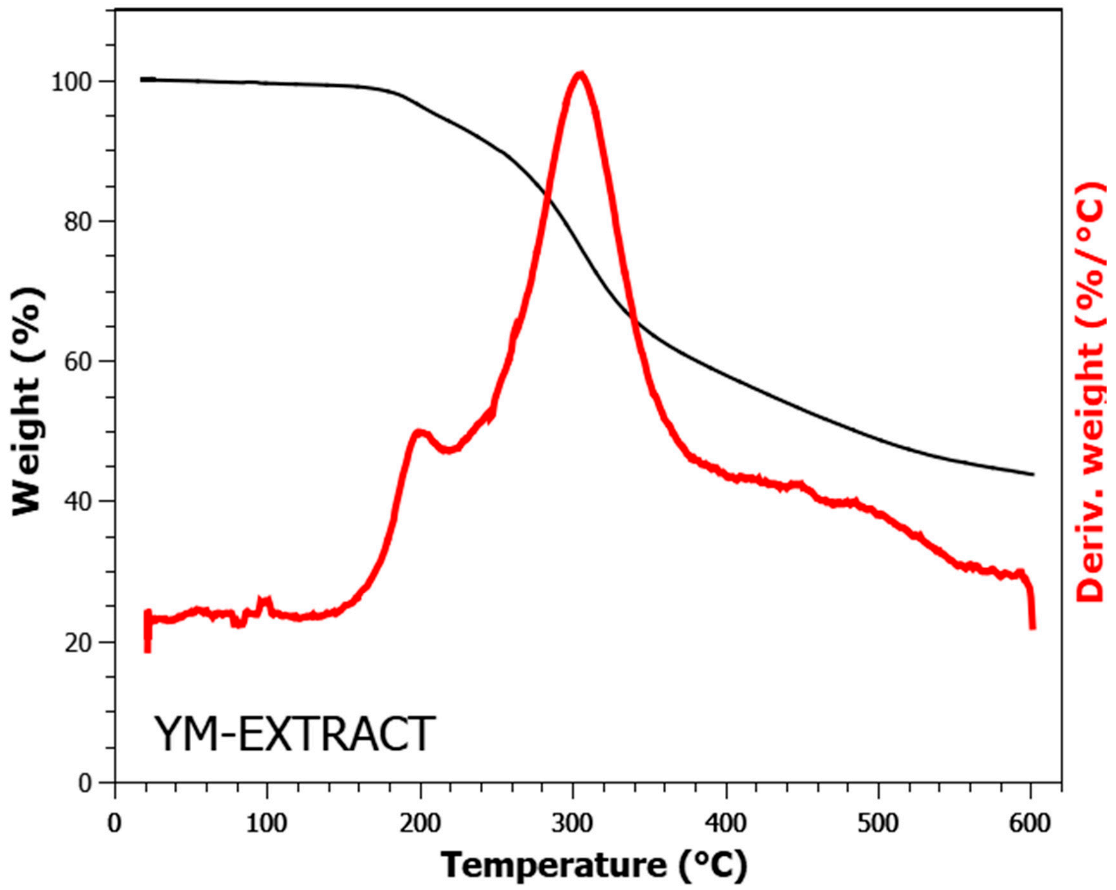

Figure S.2. Thermogravimetric analysis with its first derivative for the yerba-mate extract.

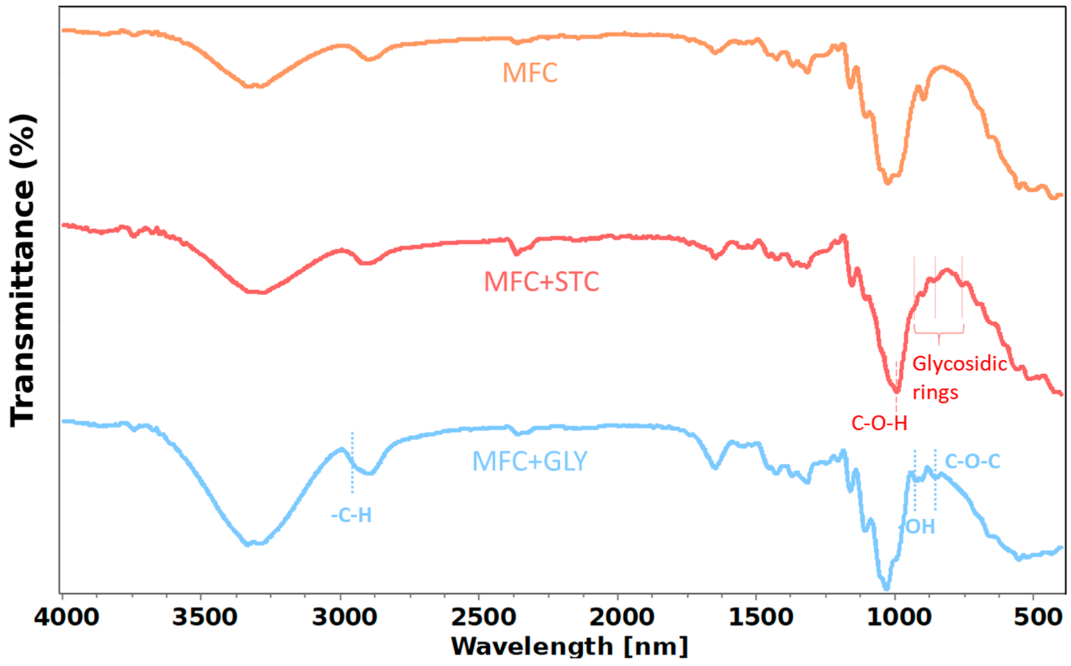

Figure S.3. FTIR of the raw materials, pure MFC, MFC blended with starch (MFC+STC) and MFC blended with glycerine (MFC+GLY).

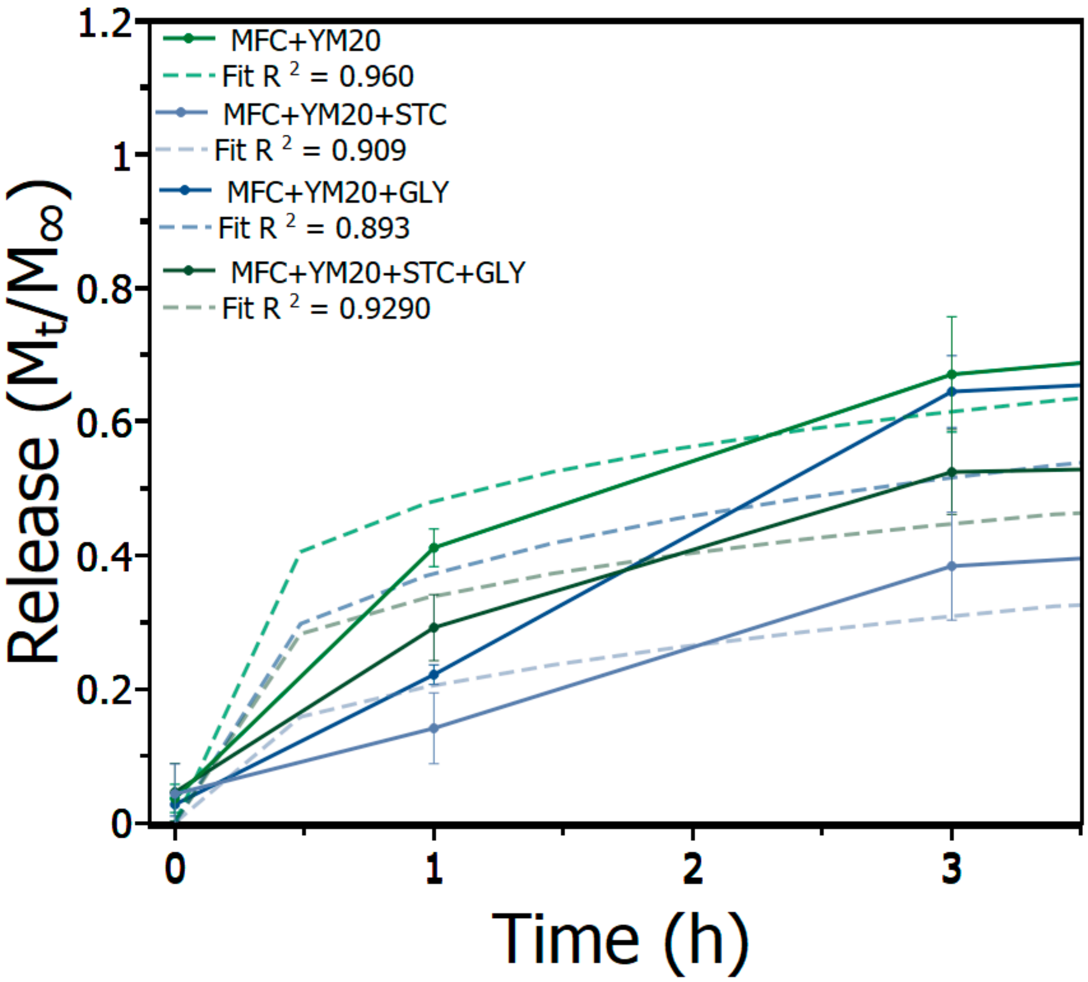

Figure S.4. Yerba mate extract release in the initial release of three hours with best-fit using Korsmeyer-peppas correlation for each curve.

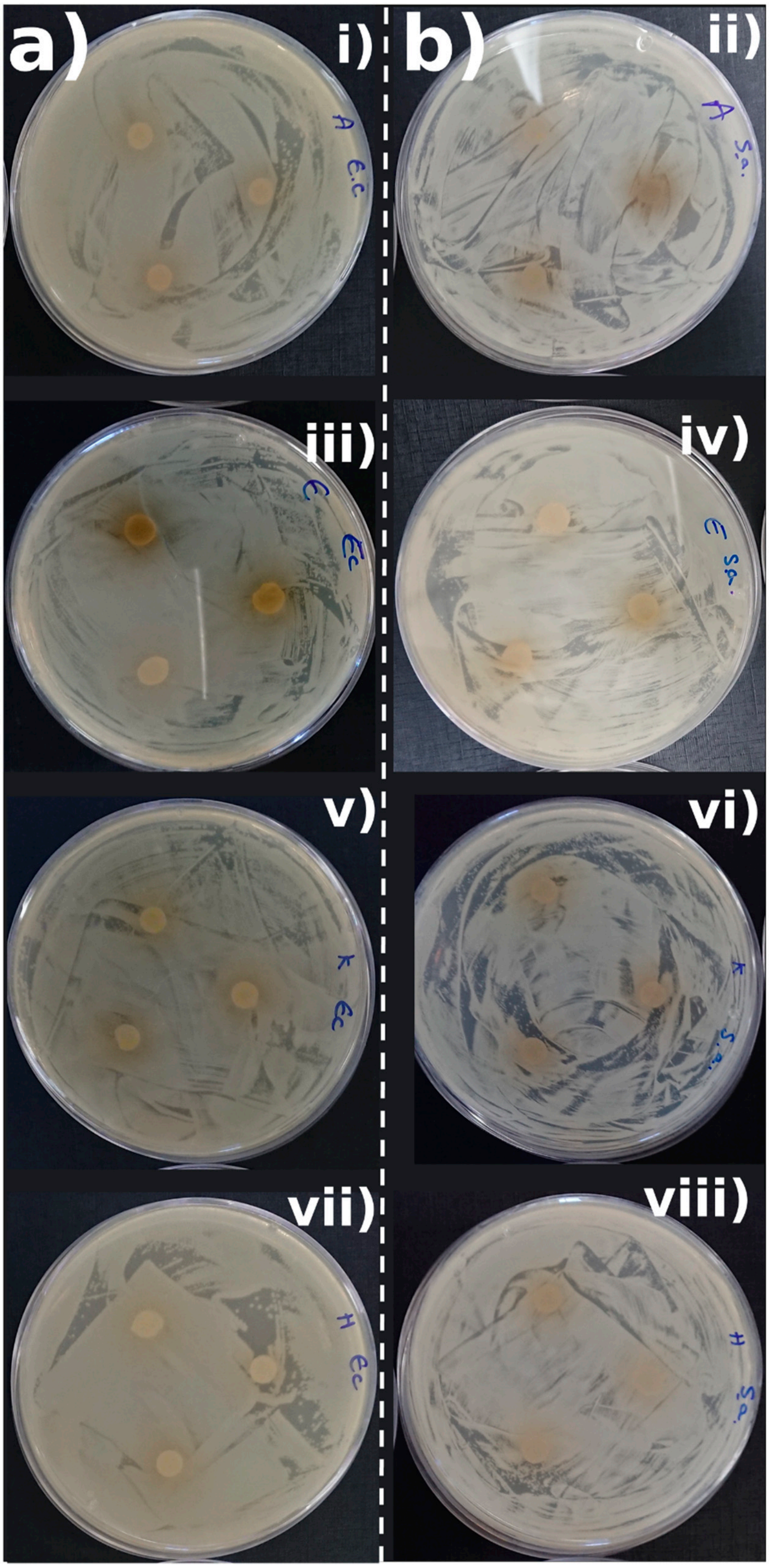

Figure S.5. Antimicrobial disk diffusion test macroscopic images for bacteria inhibition after 24 h for *S. aureus* (a) and *E. coli* (b) where (i,ii) MFC+YM20, (iii,iv) MFC+YM20+STC, (v, vi) MFC+YM20+GLY, (vii, viii) MFC+YM20+STC+GLY

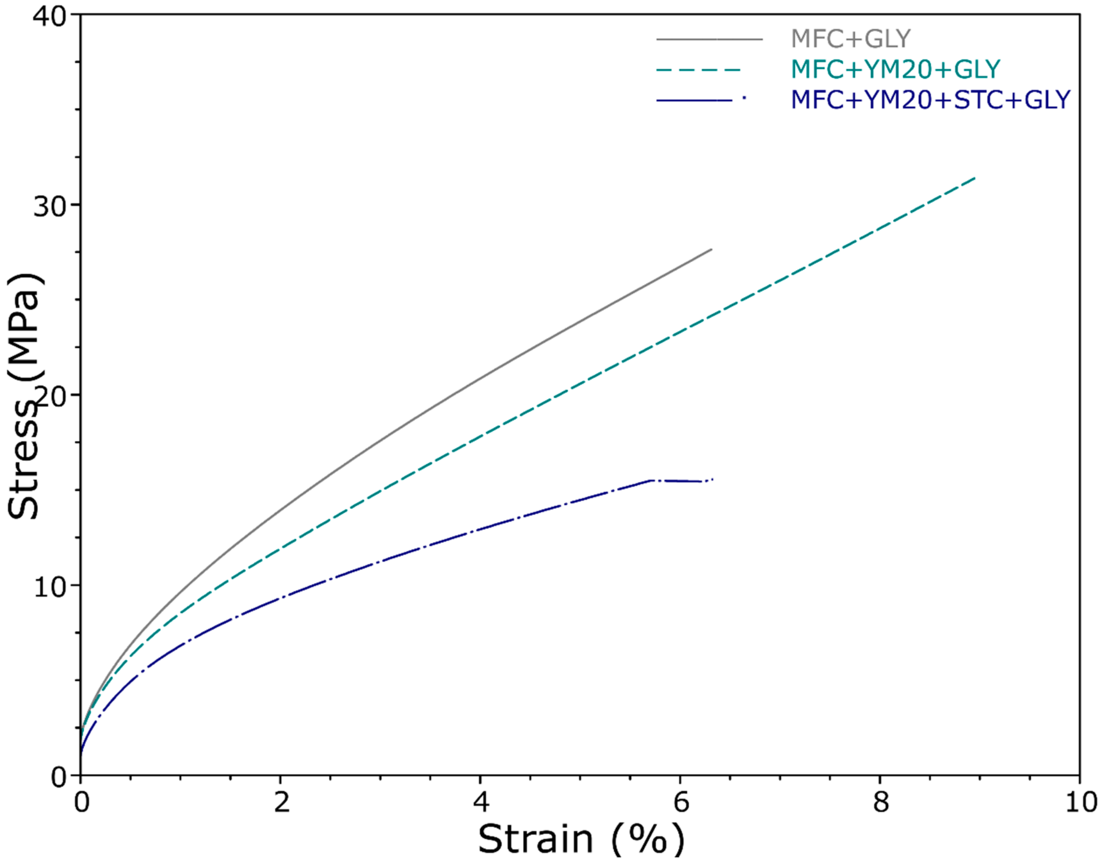

Figure S.6. Stress – strain for the MFC films containing glycerine by DMA.
